# Immunity to Polyomavirus BK Infection: Immune Monitoring to Regulate the Balance between Risk of BKV Nephropathy and Induction of Alloimmunity

**DOI:** 10.1155/2013/256923

**Published:** 2013-08-13

**Authors:** Patrizia Comoli, Michela Cioni, Sabrina Basso, Chiara Gagliardone, Leonardo Potenza, Enrico Verrina, Mario Luppi, Marco Zecca, Gian Marco Ghiggeri, Fabrizio Ginevri

**Affiliations:** ^1^Pediatric Hematology/Oncology, Fondazione IRCCS Policlinico San Matteo, 27100 Pavia, Italy; ^2^Fondazione Malattie Renali del Bambino, Istituto G. Gaslini, 16147 Genoa, Italy; ^3^Section of Hematology, Department of Medical and Surgical Sciences, University of Modena and Reggio Emilia, 41124 Modena, Italy; ^4^Pediatric Nephrology, Istituto G. Gaslini, 16147 Genoa, Italy

## Abstract

Polyomavirus BK-associated nephropathy (PyVAN) is the main infectious cause of allograft damage after kidney transplantation. A number of studies revealed an association between the presence of BKV-specific cellular immunity and BK viral clearance, with patients failing to recover specific T cells progressing to PyVAN. Evolution to allograft dysfunction can be prevented by restoration of BKV-specific immunity through a stepwise reduction of maintenance immunosuppressive drugs. Prospective monitoring of BK viral load and specific immunity, together with B-cell alloimmune surveillance, may allow a targeted modification/reduction of immunosuppression, with the aim of obtaining viral clearance while preventing graft injury due to deposition of *de novo* donor-specific HLA antibodies and late/chronic antibody-mediated allograft injury. Innovative, immune-based therapies may further contribute to BKV infection prevention and control.

## 1. Introduction

The morbidity and mortality of viral infections are significantly increasing in transplant patients. The reason resides in the severe impairment in immune surveillance caused by the development of potent induction and maintenance immunosuppressive protocols, which has led to a significant amelioration of graft outcome but, on the other hand, has weakened protective immune functions against pathogens [1]. Systematic immune control is needed in order to restrict the rate and level of latent virus reactivation since, by definition, clearance from the host cannot be obtained for such viruses, regardless of the antiviral treatment. 

Polyomavirus BK (BKV), first isolated in the 1970s, is a double-stranded DNA virus with a genome structure consisting of the early nonstructural genes encoding large T and small t antigens, the late genes encoding the capsid proteins (VP1, VP2, and VP3) and the agnoprotein and a noncoding control region (NCCR) harboring viral promoters and the origin of replication [2]. BKV seroprevalence exceeds 90% in the adult population worldwide, but the infection does not cause illness in healthy individuals [3]. Prevalence and level of BKV replication in urine, occasionally observed in healthy individuals [4], may increase with pregnancy, kidney disease, and immunodeficiency status including hematopoietic stem cell and renal transplantation [2]. In the latter setting, BKV has emerged in the last 15 years as the most challenging infectious cause of renal allograft dysfunction and graft loss [5]. BKV-related nephropathy (PyVAN) was initially reported to cause graft loss in 10% to >80% of cases [5–8], but implementation of BKV monitoring strategies after transplantation and prompt/preemptive therapeutic intervention had a positive impact on graft outcome [6, 9, 10].

In association with viral load determination, quantification of the specific immune response has gained consideration as a useful tool in the management of viral infections in the immunocompromised host. In detail, viral immunity monitoring has allowed the characterization of subgroups of patients at high risk for disease development [11] and assessment of response to antiviral therapy [12]. In addition, as control of infection depends on the restoration of a protective immune response, characterization of specific viral immunity has facilitated development of recombinant or cellular vaccines [13–15]. 

Here, we will review available evidence on BKV-specific immune responses, suggest an immunological monitoring approach to the management of BKV reactivation and PyVAN, and discuss possible future immune-based therapeutic options.

## 2. Diagnosis and Monitoring of BK Infection

PyVAN diagnosis is made by renal biopsy, with evidence of polyomavirus cytopathic changes and interstitial nephritis [5, 9, 10, 16], but the focal nature of the disease and the possible overlap with other pathologies that complicate the posttransplant course could make difficult an early diagnosis. 

PyVAN represents a complication linked to high-rate virus replication in the grafted kidney [2, 17]. Thus, monitoring of BK viruria, generally by urine cytology or quantitative PCR for viral DNA, and monitoring of BK viremia, by quantitative PCR, allow the identification of patients at risk of developing PyVAN [5, 9, 10]. Urine and plasma seem to be separate replication compartments, with plasma being directly linked to graft replication [18]. Consequently, sustained detection of BKV replication, assessed as plasma loads by quantitative PCR, is the most predictive assay for the presence of “presumptive” PyVAN [2, 5, 17], and for this reason, it is recommended by current guidelines as the best assay to guide preemptive interventions [5, 9, 10, 19–21].

In association with viral molecular monitoring, analysis of specific immune responses could become instrumental in assisting the surveillance and treatment of kidney recipients with BKV replication and PyVAN [22, 23]. However, in order to reach this aim, assessment of the most cost-effective immune monitoring protocol together with development and standardization of “high throughput” assays is needed [24].

## 3. Immune Responses in Patients with BK Infection and Disease

To introduce a proposal for a protocol of specific immune monitoring to be employed in patients with BKV replication or PyVAN, we shall give a preliminary overview of humoral and cellular immunity in patients with active BKV infection and disease.

### 3.1. Innate Immune Responses

The host immune response is of central importance in limiting primary viral infection and in controlling the virus carrier state. In general, the first line of defense against infection, prior to increase in antibody titers and epitope-specific HLA-restricted T-cell populations, is under control of innate responses and nonspecific cytotoxic cells (natural killer cells, lymphokine-activated cells, and MHC-unrestricted *γδ* ^+^ T-lymphocytes). One study demonstrated an association between lack of the HLA-C7 allele and sustained BK viremia [25], indirectly suggesting a role for inhibitory and activating killer-cell immunoglobulin-like receptors (KIRs) in the control of BKV infection. However, KIR genotyping studies, which had demonstrated a role for activating KIRs in the control of CMV infection in both hematopoietic stem cell and kidney transplantation [26, 27], ruled out an effect of KIR genotype on the rate of BKV reactivation [27].

Innate immunity may also have a detrimental role in BKV-related disease. A recent report demonstrated in biopsies with PyVAN that the activation of innate immune defense mechanisms, especially via TLR3, is implicated in the antiviral and inflammatory response [28].

### 3.2. Humoral Immune Responses

Humoral immunity still has a controversial role in the regulation of BKV activity. BKV-specific antibodies are present in 82% of individuals [4]. Studies conducted in pediatric kidney transplant recipients have shown that BKV seronegativity correlates with a higher risk of BKV replication and PyVAN [29, 30]. However, clinical observations indicate that having experienced a humoral response does not give full protection from post-transplant reactivation of viral replication and development of polyomavirus-related disease [5, 20]. 

In cohorts of kidney transplant recipients experiencing high-levels of viruria or viremia, compared to patients without active viral replication, the course of BKV-specific antibody responses has been shown to follow the level and duration of BKV replication [20, 25, 31, 32]. 

### 3.3. Cellular Immune Responses

The coincidence of PyVAN with the widespread clinical application of potent triple immunosuppressive regimens suggests a role for marked cellular immunodeficiency in disease progression. Likewise, one study showed that pretransplant dendritic cell deficiency, leading to diminished antigen presentation and T-cell activation, correlated with a high risk of posttransplant BKV viremia and progression to PyVAN [33]. Early studies demonstrated that control of BKV replication and PyVAN in kidney recipients correlated with emergence of BKV-specific cellular immune responses [34, 35]. Moreover, a longitudinal analysis showed that kidney recipients with BKV reactivation had undetectable levels of BKV-specific IFN-*γ* secreting cells. Upon immunosuppression reduction, while BKV loads in plasma and urine declined, an increase in the frequency of virus-specific T cells was observed, which coincided with reduction of serum creatinine levels, an index of allograft function stabilization [20]. A recent study confirmed that patients with self-limited BKV reactivation were those who rapidly developed BKV-specific T cells without therapeutic interventions [36].

The cellular response pattern to BKV antigens has been an object of study. Results obtained in healthy individuals and in kidney transplant recipients revealed responses to BKV large T, small t, VP1, VP2, and VP3 proteins, but no immunodominant antigen was identified [37, 38]. Analyzing cases of resolved PyVAN/past BK viremia or patients with transient/no BKV infection, two independent groups found higher frequencies of IFN-*γ* producing T cells directed to viral capsid antigens, respectively, VP1 and VP3, in the former group [20, 35, 39, 40]. In conclusion, although recent studies confirmed that lymphocytes directed to all five BKV proteins are potentially inducible [15, 38–40], the magnitude of the capsid protein-specific pool is highest. Conversely, Leuenberger et al. demonstrated that agnoprotein is immunologically ignored [41]. Cioni et al. have recently investigated whether agnoprotein might contribute to BKV immune evasion by interfering with HLA surface expression or peptide presentation and found that no HLA-ABC or DR downregulation, as well as no interference with peptide-dependent cytotoxicity, was mediated by the agnoprotein [42].

Although data are available on the distribution and immunodominance of responses to BKV proteins, there is limited evidence on their respective role in the containment of BKV replication and progression to disease. In this regard, Comoli et al. [43] have shown in a prospective study that the presence of large T antigen-specific, but not VP1-specific, cytotoxic T cells at an early time after transplantation protects from the risk of BK viruria and that in patients with viruria, the emergence of BKV LT antigen-specific immunity is associated with protection from development of BK viremia. 

Though several studies focused on BKV-specific cell immunity, it remains unclear whether BKV responses are mediated preferentially by CD4^+^ or CD8^+^ T-lymphocytes, and in particular which subset plays a protective role in the control of the infection [20, 35, 38–40]. It was shown that in kidney transplant patients with active or past BKV replication VP1-specific IFN-*γ*-producing T cells were preferentially CD4^+^, whereas the CD8^+^ population was predominantly large T specific [35]. In agreement with these observations, it was shown that BKV seropositive donors mount a powerful CTL response towards epitopes encompassed by a highly phylogenetically conserved region of the LTag implicated in viral replicative activity and in the p53-mediated control of the cell cycle of host cells [44], and BKV-directed cytotoxic activity in kidney recipients after viral clearance was mostly directed against LT antigen [20]. On the other hand, the magnitude of memory multifunctional CD4^+^ T cells was found to correlate with the severity of the previous BKV infection [40], and a higher frequency of BKV large T-specific CD4^+^ cells characterized by a cytotoxic profile (secretion of TNF-*α*, IFN-*γ*, and presence of Granzyme A and B) was shown in healthy individuals [38]. Although this issue needs to be further investigated, there is preliminary evidence suggesting that CD4^+^ T-lymphocytes could play an essential role not only in providing helper functions but also as effectors able to exert direct control of virus replication [29].

## 4. Management of BKV Reactivation, PyVAN, and the Risk of Antibody-Mediated Graft Damage: The Role of Immune Monitoring

No specific antiviral therapy has, so far, proven effective in containing PyVAN and preventing allograft damage [45, 46]. However, a number of studies have shown that progression to PyVAN can be safely prevented if BKV viremia is used to guide therapeutic intervention [17, 19, 20]. 

BKV replication is generally an early event after allografting, and therapeutic reduction of immunosuppressive agents in this crucial phase of transplantation may induce acute rejection episodes, or, worse, affect long-term allograft outcome. Indeed, in a study of PyVAN surveillance and preemptive therapy, for adult kidney recipients with BK viremia treated with concomitant reduction of calcineurin inhibitors (CI) and mycophenolate mofetil (MMF), an acute rejection incidence of 13% was observed after modulation of immunosuppression [21]. Moreover, in the cohort treated with stepwise reduction of immunosuppression by Brennan et al. [19], despite the low acute rejection rate, a significantly worse graft outcome for BK viremic patients was observed at long-term followup [47]. 

Late/chronic active antibody mediated rejection (CAMR) has emerged as an important cause of late kidney transplant failure [48]. Lately, development of *de novo* donor-specific HLA antibodies (DSA) was found to be associated with CAMR and poor graft outcome in adult and pediatric cohorts of kidney recipients at low immunological risk [49, 50]. In a low-risk pediatric population on conventional CNI-based triple drug regimen, development of *de novo* DSA occurs in almost a fourth of the patients [50]. It has been hypothesized that a potentially self-limiting and transient broad pan-B cell activation, due to non-specific stimuli [51], or to low-level allo-specific T-cell help to B-cells promoted by the loss of induction therapy effect and by protocol decrease in maintenance immunosuppression, may, under particular clinical conditions, such as molecular mimicry elicited by viral reactivation [52], be amplified and lead to the emergence of DSA [50]. In this scenario, BKV reactivation could represent both a trigger for B cell activation, and, through specific therapeutic reduction of immunosuppression, sustainment of DSA formation. 

In the setting thus delineated, detection of BKV-specific T cells by immune monitoring represents a unique tool to allow for a cautious modulation of immune suppression, with the ultimate goal of controlling viral reactivation while maintaining adequate immune suppression to protect the graft [24]. Indeed, we have evidence that as soon as virus-specific T-cell responses appear, the patient acquires protection from PyVAN progression [20]. Thus, we propose a combined immune surveillance approach: through DSA monitoring, we could identify viremic patients at potential risk of CAMR secondary to immunosuppression reduction, while BKV cellular immunity monitoring could tell us in which of these patients we may safely consider restoring part of the preinfection maintenance immunosuppression.

## 5. A Launch into the Future: Innovative Immune-Based Therapeutic Strategies to Control Polyomavirus Infection and Prevent Related Disease

As several reports have demonstrated that an efficient BKV-specific T-cell response is crucial for control of viral replication and prevention of progression to PyVAN, the development of alternative therapeutic approaches aiming to restore an effective viral-specific immune response, is an attractive alternative to current treatment options [15, 20, 34–40].

Antiviral cell therapy strategies, at first successfully employed in the setting of hematopoietic stem cell transplantation [53, 54], have subsequently been transferred to the setting of organ transplantation [55, 56]. Our group described a method for the generation of BKV-specific CTLs from BKV-seropositive healthy donors and kidney transplant patients, based on the stimulation of PBMC with dendritic cells pulsed with inactivated BKV, in the presence of IL-7 and IL-12 [57]. As result, it was possible to obtain BKV-specific T cells with cytotoxic activity. In particular, a high frequency of CD3^+^/TCR*γδ* ^+^ cells was observed, displaying an MHC-unrestricted cytotoxicity and suggesting a protective role in the control of the virus from the graft [9].

The use of BKV protein peptide mixtures as the antigen stimulus would permit good manufacturing practice (GMP) generation of T-cell lines with multiple specificities to be used for patients of any HLA type. Recently, Blyth et al. [14] generated BKV-specific T cells for possible use in adoptive cell transfer by antigenic stimulation with 15 mer peptide pools derived from VP-1, VP-2, VP-3, small t, and large T antigens. As the frequency of T cells specific for BKV was rather low when compared to other persistent viruses, 15 mer peptides may not be an optimal antigen for T-cell generation. Unfortunately, HLA-restricted T-cell response to BKV is so far poorly characterized and only few VP-1 and large T antigen epitopes have been defined, with most of the described epitopes being HLA A*02 restricted [38, 44, 58–61]. Recently, a large T antigen-derived peptide has been described that seems to elicit a CD4^+^ T-cell response across different HLA types [62].

No clinical trial has so far been published on adoptive BKV-specific T-cell transfer. However, our group was successful in treating an HSCT recipient affected by progressive multifocal leukoencephalopathy with polyomavirus JC-specific T cells obtained by stimulating PBMCs from the HSCT donor with a pool of 15 mer peptides spanning the whole sequence of JCV VP-1 and the large T antigen [63]. A similar strategy could be employed in case of JC-mediated PyVAN [64] and be translated to the treatment of BK PyVAN.

Finding immunodominant peptides would be of interest also in the context of vaccine development. As peptide vaccination is generally a poor inducer of cellular immunity, the use of carrier adjuvants may be necessary. Virus-like particles (VLPs) are optimal carrieres for antigen delivery. Murine polyomavirus VLPs were tested in mice and found to induce a strong humoral and cellular immune response [65]. An infectious recombinant BK virus, in which the large T sequence could be modified to avoid any safety implication related to the potential tumorigenicity of large T antigen, was recently proposed as an alternative strategy for vaccination in the context of BKV [66]. 

## 6. Conclusions

Successful clearance of BK viremia and prevention of PyVAN after kidney transplantation rely on the efficiency of the immune system and, more precisely, specific T-cell immunity. Reduction of maintenance immunosuppression is able to restore protective immunity, but recent evidence indicates how immune suppression modulation needs to be carefully balanced against the risk of inducing *de novo* DSA and CAMR.

Combined monitoring of DSA and BKV-specific T cells could provide an easy and safe tool for the therapeutic management of kidney recipients developing BKV reactivation. Novel immune-based strategies, including vaccination and cell therapy, might further contribute to the prevention of BKV infection and related disease.
